# First Pediatric Exercise Oncology Congress (PEOC)

**DOI:** 10.3390/curroncol30050357

**Published:** 2023-05-04

**Authors:** S. Nicole Culos-Reed, Miriam Götte

**Affiliations:** 1 Faculty of Kinesiology, University of Calgary, 2500 University Drive NW, Calgary, AB T2N 1N4, Canada; 2 West German Cancer Center, University Hospital Essen, 45122 Essen, Germany

**Keywords:** exercise oncology, pediatric oncology, implementation, research

## Abstract

On behalf of the 1st Pediatric Exercise Oncology Congress, we are pleased to present the abstracts from the 2022 Conference, the inaugural gathering of an international congress. The conference was held virtually on 7 and 8 April 2022. This conference brought together key stakeholders in pediatric exercise oncology, including multidisciplinary professionals from exercise, rehabilitation medicine, psychology, nursing, and medicine. The participants included clinicians, researchers, and community-based organizations. Twenty-four abstracts were selected for presentations (10–15-min oral presentations). In addition, there were five invited speakers with 20 min presentations and two keynotes with 45 min presentations. We congratulate all the presenters on their research work and contribution.

## 1. Abstract Themes

- Exercise interventions and physical activity recommendations
- Effects of physical activity on patient- and health-related outcomes
- Implementation of physical activity—strategies, models, considerations
- Novel methods to improve physical activity and reduce late effects
- Impacts of physical activity on cancer treatment response/tolerance
- Other topics in pediatric exercise oncology (e.g., COVID-19)

Website: https://www.pediatric-exercise-oncology-congress.org accessed on 1 March 2022

## 2. Invited Talks

### 2.1. Exercise Programs during Treatment for Hemat-Oncological Disease: Why Are Supervised Interventions Necessary Early after Diagnosis?

**Sabine Kesting ^1,2^ and Kinderklinik München Schwabing**

^1^  TUM School of Medicine, Department of Paediatrics and Children’s Cancer Research Center, Technical University of Munich, Munich, Germany
^2^  Institute of Preventive Paediatrics, Department of Sport and Health Sciences, Technical University of Munich, Munich, Germany

The adverse effects of initial anti-cancer treatment already emerge early after diagnosis and contribute to increased physical inactivity in combination with the clinical environment and psychological burden. These circumstances can instantly result in reduced physical function, impaired activities of daily living and negatively influence health-related quality of life. Thus, the question is raised: how early should we start applying exercise interventions during acute treatment and which aspects should be considered?

Experiences from a clinical perspective show a sudden interruption of normal physical activity habits immediately after hospitalization due to disease-related impairments, general uncertainty and necessary clinical diagnostics.

The approaches to implement exercise programs during this sensitive phase of treatment comprise qualified and experienced staff, individual adaption of content and intensity levels, motivating and empathetic communication, creativity and flexibility. The primary objectives are the stabilization of physical activity and fitness to reduce the risk of impairments and accustoming to the new level of physical activity and possibilities to be physically active. Despite the known challenges and difficult circumstances, exercise interventions should be applied shortly after diagnosis considering the burdensome situation and the patient’s clinical status. Moreover, key components of planning and implementing exercise early are regular communication, providing information, including the patient’s wishes and concerns, to build trust and maintain physical activity levels throughout treatment. Open research questions include the effects of medication on physical function early during treatment, e.g., initial steroid application, and suitable interventions.

### 2.2. Cardiorespiratory Fitness in Patients and Survivors

**Maxime Caru**

Department of Pediatrics, Division of Hematology and Oncology, Penn State College of Medicine, Hershey, PA, USA

Children’s and adolescents’ exposure to chemotherapeutic agents causes several long-term adverse effects affecting their cardiac health due to cardiotoxicity, physical function, and ultimately their quality of life. Physical activity is an effective strategy to prevent long-term adverse effects whether during or after treatments in pediatric oncology. Nevertheless, very few pediatric patients diagnosed with cancer meet the physical activity guidelines. Hence, less than one in two patients are doing 60 min or more of moderate-to-vigorous-intensity physical activity each day. Considering that they are physically inactive and exposed to chemotherapy-related cardiotoxicity, this has the consequence of reducing their cardiorespiratory fitness (aerobic capacity). This invited talk gives an overview of the cardiorespiratory fitness state of children and adolescents diagnosed with cancer and its long-term impact on their survivorship. In the end, the audience should be able to understand the mechanism behind the decrease in childhood cancer survivors’ cardiorespiratory fitness, identify what is needed next, and recognize clinical perspectives for this unique population.

### 2.3. Training to Support Physical Activity Delivery in Pediatric Oncology

**Amanda Wurz**

School of Kinesiology, University of the Fraser Valley, Abbotsford, BC, Canada

Qualified exercise professionals (QEPs) play a vital role in physical activity delivery, yet there are no agreed-upon training requirements to ensure the competence of PQEPs within pediatric exercise oncology. This talk will discuss this gap, provide a case example of a QEP training protocol, and offer an opportunity for critical dialogue. Ultimately, it is hoped this talk will be a first step towards creating standardized QEP training requisites in this field.

### 2.4. The Burden of Cardiovascular Disease in Childhood Cancer Survivors, Its Prevention and Management

**Christina Schindera**

No abstract available.

### 2.5. Psychosocial Empowerment through Peer Involvement in Exercise Programs

**Martin Kaj Friedh**

No abstract available.

***Abstract Oral Presentations***

### 2.6. A Bout of High-Intensity Interval Training (HIIT) in Children and Adolescents during Acute Cancer Treatment—A Feasibility Study (NOTE: Young Investigator Award Winner)

**Peter Weeber ^1^, Sabine Kesting ^2,3^, Martin Schönfelder ^1^, Henning Wackerhage ^1^ and Irene von Luettichau ^2^**

^1^  Exercise Biology, Department of Sport and Health Sciences, Technical University of Munich, Munich, Germany
^2^  Kinderklinik München Schwabing, TUM School of Medicine, Department of Paediatrics and Children’s Cancer Research Center, Technical University of Munich, Munich, Germany
^3^  Institute of Preventive Paediatrics, Department of Sport and Health Sciences, Technical University of Munich, Munich, Germany

**Background:** Low-and moderate-intensity exercise is safe and feasible during childhood cancer treatment. However, is this also true for a bout of high-intensity interval training (HIIT)? The aim of this study is to find out whether HIIT is feasible in childhood cancer patients and to measure the cardiovascular and metabolic response to HIIT.

**Methods:** Children with lymphoma, leukaemia, rhabdomyosarcoma, nephroblastoma, and synovial sarcoma performed ten 15 s high-intensity intervals (>90% of estimated HRmax) with 1 min active recovery on a bicycle ergometer in between. In addition to assessing the safety and feasibility of HIIT, we also recorded the perceived exertion rate, heart rate, lactate, and adrenaline concentrations. 

**Results:** A total of 11 patients at the age of 13.9 ± 3.6 years (*n* = 7 female) completed a bout of HIIT without serious adverse events. The patients reached a BORG value maxima of 16 ± 1.2 during exercise and heart rate increased from 78 ± 17 bpm at rest to 178 ± 12 bpm after exercise (90 ± 6% of the predicted maximal heart rate). The power-to-weight average ratio during exercise was 2 ± 0.5 W/kg. Blood lactate concentrations increased from 1.09 ± 0.50 mmol/L at rest to 5.05 ± 1.88 mmol/L post-exercise. Adrenaline levels increased only marginally post-exercise. No adverse events were observed. 

**Conclusions:** Our study shows that HIIT is feasible for a small number of physically fit childhood cancer patients but not for patients with a low fitness level or complications.

### 2.7. Maximal Cardiac Output and Cardiorespiratory Fitness in Young Cancer Survivors

**Marcella Burghard ^1,2^ and Tim Takken ^1,2^**

^1^  Child development and Exercise Centre, Wilhelmina Children’s Hospital, University Medical Centre Utrecht, Utrecht, The Netherlands
^2^  Sports and Exercise Center, Princess Máxima Center for Pediatric Oncology, Utrecht, The Netherlands

**Background**: It is known that cardiorespiratory fitness (CRF), measured as oxygen uptake relative to body weight (VO_2_peak/kg), is often decreased in both pediatric and adult cancer survivors. Research is unambiguous regarding a lower cardiac output as a possible cause for this impairment. Our aim was to assess if, next to CRF, there are differences in maximal cardiac output index (COI) response, including stroke volume index (SVI) and heart rate (HR) at maximal exercise, compared to healthy controls. 

**Methods**: A total of 30 young cancer survivors (mean age 14.4 ± 4.1 years) and 16 healthy controls (mean age 14.7 ± 4.5 years) performed a cardiopulmonary exercise test to measure CRF and other CPET-derived variables. COI and SVI were measured using thorax impedance measurements (Physioflow^®^).

**Results**: CRF (VO_2_peak/kg in percentage predicted) was significantly lower (*p* = 0.000) in the young cancer survivors (71.5; 63.5–82.6%) compared to the healthy controls (90.0; 84.0–95.0%). COI (*p* = 0.170) and peak HR (*p* = 0.914) did not differ significantly between groups, however SVI was significantly lower (*p* = 0.013) in the cancer survivor group (52.2 ± 12.8 mL/m^2^) compared to the healthy control group (61.6 ± 9.0 mL/m^2^). 

**Conclusions**: CRF is decreased after cancer treatment in young cancer survivors. A decreased cardiac stroke volume might be one of the limiting factors for CRF. 

### 2.8. Implementation of an Exercise Program for Advanced Pediatric Cancer Patients 

**Ronja Beller, Gabriele Gauß, Dirk Reinhardt and Miriam Götte**

Department of Pediatric Hematology/Oncology, Center for Child and Adolescent Medicine, Clinic for Pediatrics III, West German Cancer Centre, University Hospital Essen, Essen, Germany. 

**Background:** Children and adolescents should have access to exercise programs throughout cancer trajectory, including during palliative care. There is only very limited experience and data available for this phase of life. An exercise project has been conducted at the University Hospital Essen for advanced cancer patients who participated in supervised exercise programs during neoadjuvant and/or adjuvant treatment, and have bonded with these programs and their exercise professionals. 

**Methods and Results:** Inclusion criteria are diagnosis of advanced cancer, age >3 years, previous participation in the hospital’s exercise program, and residence in the surrounding area of the hospital (<60 min car drive). Inclusion is discussed with the Specialized Outpatient Palliative Care Team of the clinic to consider the health status and family peculiarities. After deciding on the feasibility and gaining the acceptance of the families, the assigned exercise physiologist offered sessions once a week for 30–90 min. Sessions were usually scheduled at the patients’ homes, but also in in- and out-patient clinics if patients had appointments or complications. The contents were a combination of strength, endurance, coordination, body awareness, and mobility training that were resource-oriented, related to the child’s interests, and adapted to their respective daily condition. Data monitoring was conducted every four to six weeks. 

**Conclusions:** This concept takes into account the individual possibilities, goals, and interests of those directly affected. Since the project is not yet part of standard care, it should be evaluated and expanded. Pediatric advanced cancer patients should have the opportunity to participate in exercise programs, and benefit from physical activity support. 

### 2.9. Implementation of a Pediatric Exercise Oncology Program in Brazil: Maple Kids Experience 

**Alice Aparecida Rodrigues Ferreira Francisco ^1,2^ Jader Brito Ramos dos Santos ^1^, Otávio Augusto Soares Machado ^1,3^ and Karen W. Wonders ^1,2^**

^1^  Maple Tree Cancer Alliance Brazil, Sorocaba, Brazil
^2^  Maple Tree Cancer Alliance, Dayton, OH, USA
^3^  School of Physical Education from YMCA, Sorocaba, Brazil

**Background**: Research has already shown that physical activity (PA) during childhood means a higher probability of maintaining active lifestyles. Therefore, it diminishes the severity of the adverse effects of cancer treatment and is a safe tool. Maple Tree Cancer Alliance (MTCA) is a non-profit organization helping cancer patients through exercise rehabilitation since 2011. Founded by Dr. Karen Wonders, in 2019 MTCA started serving patients in Brazil. Maple Kids (MK) started in 2021 in Brazil at the Childhood Cancer Research and Assistance Group Hospital (GPACI), a center attending 48 cities in the Sorocaba region. 

**Methods**: MK is an exercise program for pediatric cancer patients, to improve quality of life and overall health. We also include family participation, inspiring a healthy lifestyle for all. The main program goals are: to improve cardiac and pulmonary capacity, boost immune system, reduce fatigue, and peripheral neuropathy. Its secondary goals are to reduce psychological and cognitive problems, and to reduce second tumors and chronic diseases. 

**Results**: Maple Kids starts in January 2022, and results will soon be posted. 

**Conclusions**: Some children face debilitating physical and cognitive problems as side effects of cancer treatments. Cancer can mean not running or jumping, and feeling uncomfortable during PA. There is a lack of accessible and specialized programs, of staff prepared to support cancer patients, and a lot of misinformation from family and health professionals, which means that 62% of adult survivors have at least one chronic health condition. PA for pediatric cancer patients and survivors means better physical and psychosocial health.

### 2.10. Adoption of an Active Lifestyle Post Cancer Treatment: Development and Efficiency of a Behavioural, Web-Based Intervention

**Helena Koine ^1^, Isabell Hamm ^1^, Anja Chevalier ^2^ and Anna Vogelsang ^3,4^**

^1^  Institute of Psychology, German Sport University Cologne, Cologne, Germany
^2^  Department of eHealth and Sports Analytics, Ruhr-University Bochum, Bochum, Germany
^3^  Department of Sport Economics and Sport Management, German Sport University Cologne, Cologne, Germany
^4^  Faculty of Humanities, MSH Medical School Hamburg, Hamburg, Germany

**Background:** Adolescents and young adult (AYA) cancer survivors face an increased risk of physical, psychological and psychosocial late effects, long-term morbidity, and premature death. Despite the well-documented effectiveness of PA in mitigating treatment-related late effects, most adolescent and young adult (AYA) cancer survivors face various physical, behavioural, psychological, and educational barriers, which hinder them in adopting a physically active lifestyle. The current study takes survivors’ barriers into account and examines the effects of a four week online intervention on physical activity and social cognitive predictors of physical activity in AYA cancer survivors compared to a waiting control group. It is hypothesized that the physical activity levels and social cognitive predictors increase in the intervention as opposed to the control group. 

**Methods:** A randomized controlled trial with repeated measures including an intervention (*n* = 30) and awaiting control group (*n* = 31) was performed. The four week online intervention was developed based on the Health Action Process Approach and the Self-Concordance Model and included a baseline and post-test.

**Results:** Significant interaction effects between time and treatment revealed greater physical activity and improved volitional and generic social cognitive predictors of physical activity in the intervention group. No significant interaction effects were found in motivational social cognitive variables.

**Conclusions:** The results revealed the potential of a behavioural online intervention in fostering an active lifestyle in AYA cancer survivors. Given the high number of sedentary AYA and also childhood cancer survivors, the efficacy and suitability of behaviour, online lifestyle interventions for this target group will be discussed.

### 2.11. Moving the International Pediatric Oncology Exercise Guidelines (iPOEG) Forward: Where Do We Go from Here?

**Emma McLaughlin ^1^, Amanda Wurz ^1,2^, Colleen Cuthbert ^3,4,5^ and Nicole Culos-Reed ^1,5,6^**

^1^  Faculty of Kinesiology, University of Calgary, Calgary, AB, Canada
^2^  School of Kinesiology, University of the Fraser Valley, Chilliwack, BC, Canada
^3^  Faculty of Nursing, University of Calgary, Calgary, AB, Canada
^4^  Department of Community Health Sciences, Cumming School of Medicine, University of Calgary, Calgary, AB, Canada
^5^  Department of Oncology, Cumming School of Medicine, University of Calgary, Calgary, AB, Canada
^6^  Department of Psychosocial Resources, Tom Baker Cancer Centre, Cancer Care, Alberta Health Services, Calgary, AB, Canada

**Background:** Following the publication of the international Pediatric Oncology Exercise Guidelines (iPOEG) in 2021, resources to support end users were developed. Collectively, these resources are referred to as the iPOEG Toolkit. To better understand the value of the iPOEG Toolkit, its dissemination, implementation, and effectiveness will be tracked and explored. 

**Methods:** A research program informed by the Knowledge to Action framework is underway. A mixed-methods approach is being used, and the RE-AIM framework is guiding the evaluation of its **reach** (e.g., the number of iPOEG Toolkit downloads), **effectiveness** (e.g., the changed physical activity levels and patient-reported outcomes among end users), **adoption** (e.g., the number of organizations using the Toolkit), implementation (e.g., the use of and modifications made to the iPOEG Toolkit), and **maintenance** (e.g., the tracking of its continued use and impact, including markers of effectiveness, over time). Data will be collected via tracking metrics (e.g., webpage views, downloads), surveys, and interviews with relevant end users. Quantitative data will be analyzed descriptively and using regression analyses as appropriate, and qualitative data will be examined using content analysis and reflective thematic analysis. 

**Results:** The iPOEG Toolkits are currently being disseminated via the Health and Wellness Lab website, emails, and social media, and data will be gathered up until 2025. 

**Discussion:** The findings from this research program will highlight if and how the iPOEG Toolkit is being used, and offer critical guidance to optimize the iPOEG Toolkit. It is hoped that this work will support the spreading of the message that it is time for children and adolescents with cancer to ‘move more’.

### 2.12. Effect of Physical Activity on Psychosocial Health among Adult Survivors of Childhood Cancer—The SURfit Study 

**Wei H. Deng ^1,2^, Simeon J. Zürcher ^3^, Christina Schindera ^4^, Ruedi Jung ^5^, Helge Hebestreit ^6^, Iris Bänteli ^7^, Katja Bologna ^8^, Nicolas von der Weid ^4,†^, Susi Kriemler ^5,†^ and Corina S Rueegg ^1,†^**

^1^  Oslo Centre for Biostatistics and Epidemiology, Oslo University Hospital, Oslo, Norway
^2^  Oslo Centre for Biostatistics and Epidemiology, Department of Biostatistics, Institute of Basic Medical Sciences, University of Oslo, Oslo, Norway
^3^  Center for Psychiatric Rehabilitation, Universitäre Psychiatrische Dienste Bern (UPD) and University Hospital of Psychiatry and Psychotherapy, University of Bern, Bern, Switzerland
^4^  Department of Pediatric Hematology and Oncology, University Children’s Hospital Basel (UKBB) and University of Basel, Basel, Switzerland
^5^  Epidemiology, Biostatistics and Prevention Institute, University of Zurich, Zurich, Switzerland
^6^  Pediatric Department, University Hospital, Julius-Maximilians University, Würzburg, Germany
^7^  Department of Psychosomatic Medicine, University Hospital and University of Basel, Basel, Switzerland
^8^  Departement of Internal Medicine, Kantonsspital St. Gallen, St. Gallen, Switzerland
^†^  These authors contributed equally to this work.

**Backgound:** Childhood cancer survivors (CCSs) are at elevated risk of experiencing fatigue, depression, and reduced quality of life. Low physical activity (PA) levels may worsen these. Very few randomized controlled trials have investigated the effect of PA on psychosocial health among CCSs. We investigated the effect of a one year individualized exercise intervention on fatigue, mental health, and health-related quality of life (HRQoL) in adult CCSs. 

**Methods:** We randomized 151 CCSs aged ≥16 years, <16 at diagnosis, and ≥5 years since diagnosis, identified through the Swiss Childhood Cancer Registry. The intervention participants received personalized exercise counselling to increase intense PA by ≥2.5 h/week for one year. Controls maintained usual PA levels. We assessed the severity of fatigue, psychological distress, and physical and mental HRQoL at baseline, 3, 6, and 12 months. Outcomes were transformed into T-scores (mean = 50, standard deviation (SD) = 10). We used generalized linear mixed-effects models with intention-to-treat (ITT, primary), as well as per protocol allocations with compliance based on self-reported PA or a cardiopulmonary fitness test. 

**Results:** The mean baseline outcomes ranged from T-score 49.8 to 52.2, of the 133 (88%) who completed the trial. ITT analyses found significantly lower severity of fatigue by T-score −3.56 (95% confidence interval (CI) −5.69 to −1.43, *p* = 0.001) in the intervention group compared to controls at 12 months. The physical component of HRQoL was significantly better than controls in the intervention group for per protocol analyses by 3.06 (95%CI 0.99 to 5.14, *p* = 0.004) and 3.54 (1.13 to 5.96, *p* = 0.004). 

**Conclusions:** Individualized exercise interventions may improve fatigue and physical HRQoL among adult long-term survivors of childhood cancer. This is despite no effects on psychological distress. 

### 2.13. Exercise Tolerance and Physical Activity Short after Intensive Treatment in Patients with Childhood Cancer

**Miek Hornikx ^1^, Anne Uyttebroeck ^2^, Deveny Vanrusselt ^2^, Charlotte Sleurs ^2^, Marc Gewillig ^3^ and Sabine Verschueren ^1^**

^1^  Department of Rehabilitation Sciences, KU Leuven-University of Leuven, University Hospitals Leuven, Leuven, Belgium
^2^  Department of Oncology, KU Leuven-University of Leuven, University Hospitals Leuven, Leuven, Belgium
^3^  Department of Cardiovascular Sciences, KU Leuven-University of Leuven, University Hospitals Leuven, Leuven, Belgium

**Background:** Patients with childhood cancer are confronted with exercise intolerance (EI (VO_2_peak < 85% predicted)) after treatment, with a detrimental effect on quality of life and mortality. Knowledge about the limiting factor(s) for this EI and its relation to physical activity (PA) is essential in order to prescribe individually tailored rehabilitation and to stimulate physical and social reintegration. 

**Methods:** A total of 41 patients with childhood cancer (13 ± 3 years; 71% boys), diagnosed with leukemia/lymphoma (61%), a solid tumor (32%) or brain tumor (7%), and who had recently finalized their oncology-related treatment, were included in the study. Patients performed a maximal symptom-limited cardiopulmonary exercise test on a treadmill (4.8 km/h; +2% elevation/min). PA was recorded with a 3-axial accelerometer (Dynaport MoveMonitor, McRoberts, The Hague), that patients wore for 7 consecutive days. Active time (standing and walking), sedentary time, and steps were withheld. 

**Results:** Exercise tolerance (VO_2_peak: 29.7 ± 7.8 mL/min/kg (67 ± 16% predicted)) was markedly reduced in patients with childhood cancer compared to healthy peers. The majority of patients were peripherally limited (83%). A cardiac limitation was present in 71% of patients and was predominantly due to a reduced oxygen pulse (97%). Hyperventilation (32%) and a ventilatory limitation (12%) were less prevalent. The PA data of 13 patients were available (Active time: 178 ± 67 min/day; sedentary time: 515 ± 113 min/day; steps: 6411 [4458–6838]). 

**Conclusions:** Exercise tolerance is markedly reduced in patients with childhood cancer shortly after intensive treatment and this is mainly caused by the deconditioning of peripheral muscles and a reduced oxygen pulse. Further research is necessary to study the link with physical activity.

### 2.14. Implementation of a Low-Cost, High Impact Pediatric Exercise Oncology Program in Tanzania

**Manuel Ester ^1,2^ and Patricia Scanlan ^2,3^**

^1^  Faculty of Kinesiology, University of Calgary, Calgary, AB, Canada
^2^  Tumaini La Maisha, Tanzania
^3^  Department of Pediatric Oncology, Muhimbili National Hospital, Tanzania

**Background**: Despite the physical and psychosocial benefits of exercise for children and adolescents living with and beyond cancer, few exercise oncology programs have been successfully implemented for this population. Implementation barriers herein include cost as well as a lack of qualified exercise professionals and appropriate facilities, which may be exacerbated in resource-limited countries. The purpose of this implementation project was to overcome these barriers and implement a sustainable exercise oncology program with Tumaini La Maisha (TLM) in Tanzania. 

**Methods**: Interdisciplinary stakeholder discussions were held between nurses, oncologists (PS), caregivers, TLM teachers, and the visiting exercise professional (ME) in order to provide a broad range of perspectives and agree upon a feasible exercise program for implementation at Muhimbili National Hospital. Cost barriers were addressed by using volunteers and paid TLM staff for program delivery, with the exercise professional educating staff on exercise principles. To address facility barriers, a public sports field served as the exercise space. 

**Results**: Discussions led to the development and implementation of a 30 min teacher-led, group- and family-focused, play-based exercise program. The program was delivered daily for 3 months, with high attendance and satisfaction from parents and children. Three TLM teachers were trained for long-term program delivery. Newly identified barriers included sun exposure risks (addressed via indoor exercise) and a lack of cultural relevancy (addressed by integrating traditional dance within the program). 

**Conclusions**: The involvement of stakeholders in the implementation planning and consideration of resource limitations led to the successful implementation and maintenance of an exercise oncology program in a resource-limited setting.

### 2.15. Network ActiveOncoKids as a National Implementation Approach for Exercise as Usual Care in Pediatric and Adolescent Oncology

**Miriam Götte ^1^, Regine Söntgerath ^2^, Gabriele Gauß ^1^, Joachim Wiskemann ^3^, Mirko Buždon ^4^ and Sabine Kesting ^5,6^**

^1^  Department of Pediatric Hematology/Oncology, Clinic for Pediatrics III, West German Cancer Centre, University Hospital Essen, Essen, Germany
^2^  Department of Pediatric Oncology, Hematology and Hemostaseology, University Hospital Leipzig, Leipzig, Germany
^3^  Working Group Exercise Oncology, Division of Medical Oncology, University Clinic Heidelberg and National Center for Tumor Diseases (NCT), Heidelberg, Germany
^4^  Institute of Sports Medicine, Hannover Medical School (MHH), Hannover, Germany
^5^  Institute of Preventive Pediatrics, Department of Sport and Health Sciences, Technical University of Munich, Munich, Germany
^6^  Kinderklinik München Schwabing, TUM School of Medicine; Department of Pediatrics and Children’s Cancer Research Center, Technical University of Munich, Munich, Germany

**Background:** Cancer and the acute and late effects of its treatment are associated with a decline of physical activity behavior in childhood cancer patients and survivors. Children have a legal right to exercise as well as to the active participation in physical activities, and to the positive effects of exercise-related benefits.

**Methods:** Network ActiveOncoKids (NAOK) is a Germany-wide initiative with the main goal of enabling children, adolescents, and young adults with exercise opportunities during and after cancer treatment. The network uses and bundles the knowledge, expertise, and experience of decentralized clinical sites and working groups on the topic of “Exercise and Sport in Pediatric Oncology”. For this purpose, NAOK was founded in 2012, striving for networking and structuring. It is managed and accompanied by an overarching coordination, a steering group, and an advisory board. Its main aims are A) physical activity support for patients and families, B) policy change to establish structures and guidelines, and C) the generating of evidence through scientific projects. 

**Results:** NAOK brings together 33 pediatric oncology treatment centers that offer exercise interventions either during acute treatment and/or in follow-up care. The main focus of the last 2.5 years was to support children, adolescents, and young adults through individualized counselling, to help in implementing exercise programs at pediatric oncology centers, and to adapt and change medical and care structures (e.g., via guidelines and educational lessons).

**Conclusions:** NAOK is an interdisciplinary network that supports the implementation of pediatric exercise oncology in usual care. In the past few years, great progress has been achieved in the areas of exercise implementation, structural organization, and communication, which might serve as a model for other countries.

### 2.16. Reh-PLAY Project: The Italian Rehabilitation Website for Children and Adolescents Affected by Oncoematological Diseases in the Pandemic COVID-19 Context

**Lucia Longo ^1^, Annalisa Cornelli ^2^, Riccardo Ruisi ^3^, Francesca Rossi ^4^, Filippo Gatti ^5^, Federica Ricci ^4^, Daniele Bertin ^4^, Giulia Zucchetti ^4^ and Franca Fagioli ^4,6^**

^1^  Associazione Unione Genitori Italiani Contro il Tumore dei Bambini ODV, Torino, Italy
^2^  Ospedale Papa Giovanni XXIII, Associazione ConGiulia ONLUS, Bergamo, Italy
^3^  Istituto Ortopedico Rizzoli, Bologna, Italy
^4^  A.O.U. Città Della Salute e Della Scienza, Presidio Ospedale Infantile Regina Margherita, Torino, Italy
^5^  Università degli Studi di Torino, Corso di Laurea in Terapia della Neuro e Psicomotricità dell’Età Evolutiva
^6^  Università degli Studi di Torino, Torino, Italy

**Background:** With the onset of the COVID-19 pandemic in Italy and its related restrictions, the National Orders Federation of Technical, Rehabilitation and Prevention Health Professions, recommended remote rehabilitation interventions. Therefore, in April 2020, the rehabilitation working group of the Italian Hematology and Oncology Association (AIEOP) created a website for telerehabilitation within the “#IoMiMuovoACasa” (#IDoHomeTraining) pilot project. According to the preliminary safety and efficacy results, and users’ opinions, the need for a more structured website arose. The Reh-PLAY project aims to create a new website to support remote individualized rehabilitation interventions, in addition to the direct treatment, for the prevention/rehabilitation of motor difficulties related to the disease and/or to treatment side effects. 

**Methods:** The website contents are written according to the AIEOP Consensus Conference on Rehabilitation in Pediatric Oncohematology. Exercise videos created by physiotherapists are based on their expertise and are smartly accessible by the professionals who can create personalized exercise programs, available in a patient’s personal area. 

**Results:** In one month, twenty patients used the website. The Reh-PLAY website is smartly accessible for families, patients, and physiotherapists from the AIEOP website. Professionals can select texts/infographics about prevention and rehabilitation, and access 250 exercise videos, divided by type, age, and intensity. Patients can download information brochures, adhesion diaries, patients reporting outcome measures, and a questionnaire to improve the service. 

**Conclusions**: Growing evidence in Pediatric Oncohematology identifies rehabilitation as a fundamental aspect of treatment. The Reh-PLAY website improves continuity in rehabilitation management, supporting families and patients with telerehabilitation, and facilitating the dissemination of knowledge between professionals.

### 2.17. Lessons Learned from Innovating the Pediatric Cancer Patients and Survivors Engaging in Exercise for Recovery (PEER) Program to an Online Modality Due to COVID-19

**Shaelene Standing ^1^, Rachel McInnes ^2^, S. Nicole Culos-Reed ^3^, Mackenzie Murawsky ^2,4^, Gregory M. T. Guilcher ^5,6^, Fiona Schulte ^5,6^ and Carolina Chamorro Vina ^2,3^**

^1^  Cumming School of Medicine, University of Calgary, Calgary, AB, Canada
^2^  Kids Cancer Care Foundation of Alberta, Calgary, AB, Canada
^3^  Faculty of Kinesiology, University of Calgary, Calgary, AB, Canada
^4^  Faculty of Health Sciences, University of Ottawa, Ottawa, ON, Canada
^5^  Department of Oncology, Cumming School of Medicine, University of Calgary, Calgary, AB, Canada
^6^  Section of Oncology/Transplant, Alberta Children’s Hospital, Calgary, AB, Canada

**Background:** The focus of the PEER program is to increase physical literacy, fitness, and to promote socialization for children affected by cancer and their siblings. In response to the COVID-19 pandemic, the program transitioned to an online format. The sessions, for children (6–11 years and 12–17 years), ran 1/week. Sport kits were provided to participants. 

**Methods:** After 12 months of running the program, which was attended regularly by 17 families (16 kids and 9 teens), we sought participant feedback through anonymous surveys. A total of 12 parent and 10 participant responses were collected outlining information about motivators for and barriers to online programming, safety, and satisfaction. 

**Results:** The families participated in the program mainly to (1) improve fitness and (2) increase peer connections. Of these, 100% felt safe, 95% agreed that sport kits increased engagement, and most agreed that sport kits also helped kids increase their activity level after participation in the PEER sessions. Furthermore, 86% felt that 45–60 min was a good session duration and that participation in the program increased their child’s fitness level. 

**Conclusions:** This program was (i) engaging, (ii) safe, and (iii) accessible for children affected by cancer who are medically vulnerable and/or remotely located. We suggest a program length of 45–60 min based on the age group. Exercises should have a greater focus on physical literacy and fundamental actions for younger age groups and should be more fitness-based for teens. Fun and the inclusion of socialization are key elements in increasing enjoyment, adherence, and community belongingness. The provision of a sport kit is recommended. Individualized adaptations are necessary for program success.

### 2.18. Promoting Positive Physical Activity Behaviours in Children and Adolescents Undergoing Acute Cancer Treatment: Feasibility of the CanMOVE Intervention

**Sarah L. Grimshaw ^1,2^, Nicholas F. Taylor ^1^, Rachel Conyers ^2^ and Nora Shields ^1^**

^1^  La Trobe University, School of Allied Health, Human Services and Sport, Bundoora, Melbourne, VIC 3086, Australia
^2^  Murdoch Children’s Research Institute, Parkville, Melbourne, VIC 3052, Australia

**Background:** Supporting children/adolescents with cancer to be more physically active has the potential to improve short- and long-term outcomes. The objective of this study was to assess the feasibility of CanMOVE, a complex, theoretically informed, behaviour change intervention to promote participation in physical activity for children/adolescents undergoing acute cancer treatment.

**Method:** A feasibility study utilising single-group, repeated measures, and a mixed methods design was completed. Participants completed the 10 week CanMOVE intervention, which involved structured support from a physiotherapist and the provision of a Fitbit (child and parent). Feasibility domains of demand, acceptability, implementation, practicality, limited efficacy, and integration were evaluated. Objective assessments of physical activity, physical function, and health-related quality of life were completed. Qualitative data were collected via semi-structured interviews with participants (parents and children/adolescents) and focus groups with clinical staff.

**Results:** Twenty families completed CanMOVE, including children/adolescents (median age 12 years, range 5–16) with a mix of cancer diagnoses. There was a high demand for CanMOVE with a 95% enrolment rate. CanMOVE was acceptable from both participant and staff perspectives. All feasibility thresholds set for implementation were met. There were no serious adverse events. Under limited efficacy, data indicate that CanMOVE shows promise in influencing child/adolescent physical activity behaviour. Positive impacts were also seen in parent and staff behaviour towards physical activity promotion. The pre/post physical function and HRQOL assessments showed positive trends. 

**Conclusions:** CanMOVE is feasible and safe to implement in the paediatric oncology setting. CanMOVE shows potential in influencing the behaviour of children/adolescents and of the people in their social and professional support networks. These findings can be used to inform services in the paediatric cancer setting to ensure physical activity promotion is a considered and prioritised aspect of clinical care.

Abstract Poster Presentations

### 2.19. Maximal Activity; Health Care Program for Children with Cancer during Hospitalization

**F V. Engels, D Agterberg, W. P. Bekkering and Patrick van der Torre**

Department of Sports and Exercise Center, Princess Máxima Center for Pediatric Oncology, Heidelberglaan 25, 3584 CS Utrecht, The Netherlands

**Background:** International studies and organizations, such as the World Health Organisation, have identified the health risks across lifespan that are associated with physical inactivity. Childhood cancer and its treatment have considerable impact on a child’s physical and mental wellbeing and often lead to reduced physical activity levels and sedentary behaviour. The combination of long-term chemotherapy, surgery and/or radiotherapy as administered in children with cancer especially impairs physical activity and fitness, both during and after therapy. Physical activities are important for the development of children and increasing evidence suggests the beneficial effects of physical activity promotion during cancer treatment as well. Therefore, ways to promote physical activity and exercise are becoming an important part of children’s cancer treatment. By means of our “Maximal Activity” program in the Princess Máxima Center for Pediatric Oncology, we want to encourage our patients to get out of bed and be as active as possible, both during and after treatment. 

**Methods:** A qualitative survey of 30 individuals (children, families and health care professionals) was used to ask about the experiences of the developmentally appropriate care program, of which ‘Maximal Activity’ is a part. 

**Results:** An important point that emerged was that the facilities and environment invite physical activity. The program was named by many as valuable. Trust and confidence in physical activity increased during hospitalization. Other results still need to be analysed and will be presented at the congress. 

**Conclusions:** Maximal Activity is a health program that contributes to a stimulating environment for physical activity and is appreciated by all stakeholders. 

### 2.20. Assessing the Physical Activity Behaviour of Parents Whose Children Have Cancer before and during Intensive Cancer Treatment

**Carolin Ohnmacht and Alexander Puzik**

Department of Pediatric Hematology and Oncology, Medical Center—University of Freiburg, Faculty of Medicine, University of Freiburg, Mathildenstraße 1, D-79106 Freiburg, Germany

**Background:** Regular physical activity (PA) is essential for biopsychosocial health, while reduced PA during childhood cancer treatment leads to increased late effects in affected children. Moreover, cancer and its treatment determine the everyday lives of families and parents who spend plenty of time with their children in hospital. Thus, it can be assumed that the parents’ PA is significantly affected during cancer treatment. Meanwhile, parents’ PA-behaviour has a pronounced influence on their children’s behaviour. The aim of this project is to investigate the parents’ PA-behaviour before and during their children’s cancer treatment. 

**Methods:** The PA and sedentary behaviour of the parents were assessed before and during their children’s intensive oncological therapy in a cross-sectional design using the International Physical Activity Questionnaire (IPAQ-SF). 

**Results:** A total of 40 parents of children with cancer took part in this survey. They were interviewed no earlier than their children’s second inpatient stay. The parents’ PA levels before diagnosis were in line with the reference values for healthy adults in Germany. During the children’s treatment, all dimensions of the parents’ daily PA, and the number of minutes of PA per week, decreased significantly (*p* < 0.001). The greatest reduction in PA was identified during inpatient stays, with a significant increase in sitting time (*p* < 0.001). 

**Conclusions:** This is the first study to show that the PA of parents whose children have cancer decreases significantly during cancer treatment. As parents’ PA behaviour significantly affects that of their children, even after completion of cancer treatment, future exercise programs in pediatric oncology should include parents in order to reduce inactivity-related late effects.

### 2.21. Get Strong to Fight Childhood Cancer: An Exercise Intervention for Children and Adolescents Undergoing Anti-Cancer Treatment (FORTEe) 

**Sandra Stöessel ^1^, Elias Dreismickenbecker ^1^, Marie A. Neu ^1^, Lisa Ploch ^1^, Norbert Paul ^1^, Christian Ruckes ^1^, Adriana Balduzzi ^2^, Francesca Lanfranconi ^2^, Peter Wright ^3^, Stan Windsor ^3^, Joachim Wiskemann ^4^, Inaam El-Rajab ^4^, Veronika Picmanova ^5^, Céline Gravot ^5^, Rodolf Mongondry ^6^, Wilhelm Bloch ^7^, Katie Rizvi ^8^ and on behalf of Youth Cancer Europe, Martin K. Fridh ^9^, Alejandro Lucia ^10^, Carmen Fiuza-Luces ^10^, Miriam Götte ^11^ and on behalf of Network ActiveOncoKids, Filippo Spreafico ^12^, Barbara Konda ^13^, Lidija Kitanovski ^14^, Lena Werthmann ^15^, Tobias Baader ^16^ and Jorg Faber ^1^ and on behalf of the FORTEe Consortium**

^1^  University Medical Center of the Johannes Gutenberg, University Mainz (DE)
^2^  Fondazione Monza e Brianza per Il Bambino e La Sua Mamma (IT)
^3^  Oxford Brookes University (UK)
^4^  Heidelberg University Hospital (DE)
^5^  Concentris Research Management GmbH (DE)
^6^  Centre de Lutte Contre le Cancer Léon Bérard (FR)
^7^  German Sport University Cologne (DE)
^8^  Youth Cancer Europe (RO)
^9^  Department of Pediatric and Adolescent Medicine, Copenhagen University Hospital, Copenhagen, Denmark (DK)
^10^  Universidad Europea de Madrid (ES)
^11^  Essen University Hospital (DE)
^12^  Fondazione IRCCS Istituto Nazionale dei Tumori (IT)
^13^  Forma 3D Ltd. (SI)
^14^  University Medical Center Ljubljana, Division of Pediatrics, Department of Haematooncology (SI)
^15^  Nurogames GmbH (DE)
^16^  Pixformance Sports GmbH (DE)

**Background:** Cancer is the leading cause of death by non-communicable diseases in children in Europe. During cancer treatment, patients’ morbidity is increased due to physical inactivity and cancer-related fatigue. Precision-based exercise training programs in children and adolescents attending the intensive phases of cancer treatment is an increasingly promising therapy. However, strong evidence for exercise efficiency is lacking in paediatric oncology and, thus, precision exercise training is not part of standard care and does not reach the majority of patients. 

**Methods:** The FORTEe project is structured in seven work packages and intends to evaluate a personalised and standardised exercise intervention in 450 children, adolescents and young adults undergoing cancer treatment in 9 centres across Europe. The randomised, controlled FORTEe trial aims to generate high evidence for an innovative, patient-centred exercise treatment. Supervised exercise training intends to impact the efficiency of systems involved in the oxidative metabolism chain, including the skeletal muscle. The tailored training is also focused on strength in order to counteract muscular atrophy. Within the project, digital and innovative technologies (a FORTEe app, an augmented reality program and an interactive digital training) will be developed and applied to make the exercise training more attractive, age-adapted and inspirational. FORTEe will stimulate multidisciplinary research by involving paediatricians and exercise scientist in order to provide more inclusive access to paediatric exercise oncology. 

**Conclusions:** Progressing beyond the current state-of-the-art standard, FORTEe has the ambition of implementing paediatric exercise oncology as an evidence-based standard in clinical care for all childhood cancer patients worldwide. 

### 2.22. Therapeutic Exercise in Children Diagnosed with Solid Tumors: A Scoping Review

**Brooke E. Kohler,^1^ Emmah Baque ^1,2^** **, Carolina X. Sandler ^3,4,5^ and Stewart G. Trost ^1,6^**

^1^  Faculty of Health, Queensland Centre for Children’s Health Research, Queensland University of Technology (QUT) Brisbane, Brisbane, QLD, Australia
^2^  School of Health Sciences and Social Work, Griffith University, Nathan, QLD, Australia
^3^  UNSW Fatigue Research Program, Kirby Institute, University of New South Wales, Sydney, NSW, Australia
^4^  School of Sport and Exercise Science, School of Health Sciences, Western Sydney University, Sydney, NSW, Australia
^5^  Menzies Health Institute Queensland, Griffith University, Griffith, QLD, Australia
^6^  School of Human Movement and Nutrition Sciences, University of Queensland, Brisbane, QLD, Australia

**Background:** Increasing survival rates for children with solid tumors present an ongoing challenge regarding how to maximize the quality of survivorship and effectively manage the short- and long-term complications of the disease and its treatment. To gain a better understanding of the research pertaining to therapeutic exercise programs, we conducted a scoping review of exercise training studies conducted in children diagnosed with solid tumors. 

**Method:** A systematic literature search was performed across four electronic databases. Articles were selected for full-text review if they included participants diagnosed with a solid tumor, if at least 50% of the participants were aged ≤21 years, if they evaluated an exercise program ≥ 2-weeks in duration, and if they were published in an English, peer-reviewed journal. 

**Results:** Of the 6648 citations identified, 14 articles met the inclusion criteria. Only three studies were conducted as randomized controlled trials. The duration of the exercise programs ranged from 3 to 40 weeks and their frequency ranged from 3 to 11 sessions per week. Exercise session duration ranged from 15 to 180 min, with most articles reporting 30–90-min sessions. A range of exercise modalities were employed, including ergometry, resistance training, yoga, and active videogaming. Adherence ranged from 77% to 100%, with no adverse events reported. Exercise training resulted in improvements in cardiorespiratory fitness, functional strength, physical activity, and quality of life. 

**Conclusions:** A small number of mostly low-quality studies have evaluated therapeutic exercise in pediatric survivors of solid tumors. Although limited, the extent of research supports the feasibility and safety of therapeutic exercise and suggests that therapeutic exercise may be potentially effective in improving a range of health outcomes. 

### 2.23. Preventing Sensory and Motor Dysfunctions in Children Receiving Neurotoxic Chemotherapy—Overview of the Current Therapy Options and Study Protocol of the PrepAIR Randomized Controlled Multi-Center Trial

**Clémentine Bischoff ^1^, Nicolas Von der Weid ^2^, Oliver Faude ^1^, Fiona Streckmann ^1,3^ and on behalf of the PrepAIR Study Group ^†^**

^1^  Department of Sport, Exercise and Health, University of Basel, Grosse Allee 6, 4052 Basel, Switzerland
^2^  Department of Pediatric Oncology and Hematology, University Children’s Hospital Basel, University of Basel, Spitalstrasse 33, 4056 Basel, Switzerland
^3^  Department of Oncology, University Hospital Basel, Petersgraben 4, 4031 Basel, Switzerland
^†^  PrepAIR Study Group: Katrin Scheinemann (KS Aarau), Uta Tacke (UKBB), Jochen Rössler (Insel Bern), Pablo Hernaiz Driever (Charité Berlin), Miriam van Buiren & Alexander Puzik (Uniklinik Freiburg, Jeanette Greiner (KISPI St. Gallen), Aline Rehm & Patrizia Brunner (Universität Basel), Valentin Benzig & Regula Everts (Universität Bern), Anja Wehrle (Universität Freiburg), Claudia Bucher (KS Aarau), Sandra Frauchiger (Insel Bern), Carolin Ohnmacht (Uniklinik Freiburg), Martina Sigg (KISPI St. Gallen).

**Background:** Modern therapy improved survival for children with cancer. However, the treatment has unintended consequences. Depending on neurotoxic agents, 52–100% of children develop a chemotherapy-induced peripheral neuropathy (CIPN). The severe symptoms such as loss of sensation, numbness, pain, absent reflexes and loss of balance control, not only delay motor development milestones such as walking, running, jumping, or climbing, diminishing children’s quality of life and affecting their social reintegration, but are also of high clinical relevance. Streckmann et al. confirmed in their meta-analysis that recovery is poor, but there is a clear benefit for adults in favor of sensorimotor training (SMT) to target the symptoms of CIPN. 

**Methods**: In our RCT, we will recruit N = 131 children from 6 centers (Switzerland and Germany). Immediately after being scheduled to receive neurotoxic chemotherapy, the intervention group will perform a standardized, age-adjusted, specific playful SMT program twice a week for the duration of their medical therapy, while the control group will receive treatment as usual. 

**Results**: For the intervention, we created a training manual which will finally lead to a simple therapy option for children suffering from CIPN. The manual provides a basis for playful SMT based on a modular system, which leaves the therapists and the children room for scope and still respects all the training modalities within the background of “specific exercise is medicine”. 

**Conclusions:** We hypothesize that children in the intervention group will develop fewer symptoms of CIPN and will be able to maintain their motor and sensory functions for an age-appropriate motor development.

### 2.24. Impact of the COVID-19 Pandemic on the Availability of Exercise Programs in Pediatric Oncology: A Survey of Providers in Germany

**Dominik Gaser ^1,2^, Christiane Peters ^2^, Renate Oberhoffer-Fritz ^2^, Miriam Götte ^3^, Gabriele Gauß ^3^, Irene Teichert-von Lüttichau ^1^ and Sabine Kesting ^1,2^**

^1^  Department of Pediatrics and Children’s Cancer Research Centre, Children’s Hospital München Schwabing, TUM School of Medicine, Technical University of Munich, Munich, Germany
^2^  Department of Sport and Health Sciences, Technical University of Munich, Munich, Germany
^3^  Department of Hematology and Oncology, Clinic of Pediatrics III, West German Cancer Centre Essen, University Hospital, Essen, Germany

**Background:** An increasing amount of in-hospital and out-patient physical activity offers facilitate access to professional exercise programs for children and adolescents during acute anticancer treatment and surveillance. The COVID-19 pandemic has presented major challenges to hospitals and rehabilitation facilities. An online survey among providers in Germany investigates the impact of the pandemic on individual sectors of exercise programs in pediatric oncology. 

**Methods:** From 19 January until 9 February 2022, all German clinics and institutions with an exercise program for pediatric cancer patients and/or survivors are invited to participate in an online survey. Methodology and recruitment is conducted in cooperation with Network ActiveOncoKids. Limitations, challenges and measures for adapting offers in the context of the individual pandemic waves, are collected. In addition, challenges for the implementation of scientific studies are analyzed. 

**Results:** Thirty-three sites have been requested to participate in the survey. We assume that exercise professionals and scientists have used the pandemic-related challenges to modify the existing concepts of exercise promotion and adapt them to the specific local conditions. 

**Conclusions:** We expect new ideas and approaches for the realization of exercise programs under pandemic conditions. The extension of digital offers may improve the access for children and adolescents to exercise programs in pediatric oncology in the long term and, therefore, could potentially be adopted into standard exercise care.

### 2.25. A Review to Map the Evidence of Physical Activity Interventions in Post-Treatment Adolescent and Young Adult Cancer Survivors 

**Maxime Caru ^1,2^, Ariane Levesque ^3^, Pooja Rao ^1^, Smita Dandekar ^1^, Christopher Terry ^4^, Valerie Brown ^1^, Lisa McGregor ^1^ and Kathryn Schmitz ^2^**

^1^  Department of Pediatric Hematology and Oncology, Penn State College of Medicine, Hershey, PA, USA
^2^  Department of Public Health Sciences, Penn State College of Medicine, Hershey, PA, USA
^3^  Department of Psychology, University of Montreal, Montreal, QC, Canada
^4^  Department of Medical Oncology, Sidney Kimmel Cancer Center—Thomas Jefferson University, Philadelphia, PA, USA

**Background:** AYA cancer survivors are a population that have unmet needs, and researchers believe that physical activity (PA) interventions can address many of these needs. This review describes and synthesizes previously reported data in order to map the current evidence as well as to identify gaps in knowledge and future research needs. 

**Methods:** A search of the literature was conducted in PubMed, CINAHL, EMBASE, Web of Science and Cochrane Library. This review followed the PRISMA-ScR statement. We included all original studies investigating PA interventions in post-treatment AYA cancer survivors who were between 15 and 39 years old at the time of their initial cancer diagnosis. 

**Results:** A total of 8 studies were included in this review and showed that PA interventions were feasible and acceptable in AYA cancer survivors. Overall, PA interventions were individualized and mainly aerobic in nature. Studies examining the effects of PA interventions on AYA cancer survivors’ health evaluated physical and mental health outcomes, including, but not limited to, physical functioning, functional capacity, occupational performance, health-related quality of life, physical capacities, fitness, mood, and self-perception after cancer treatments. 

**Conclusions:** Our review maps the current evidence of PA interventions and highlights the paucity of data in this area of investigation, obviating how much work remains to be carried out to demonstrate the potential benefits of PA on AYA cancer survivors’ health outcomes. Future studies should consider the development PA interventions that address patients’ clinical needs, in addition to providing a detailed description of PA interventions. 

### 2.26. Sensorimotor Training—Therapeutic Potentials and a Child-Specific Training Concept for Pediatric Cancer Patients

**Sarah Otten ^1^, Clémentine Bischoff ^2^, Julia Däggelmann ^1^, Fiona Streckmann ^2,3^, Wilhelm Bloch ^1^ and Vanessa Oschwald ^1^**

^1^  Institute for Cardiovascular Research and Sports Medicine, German Sport University Cologne, Am Sportpark Müngersdorf 6, 50933 Cologne, Germany
^2^  Department of Sport, Exercise and Health, University of Basel, Birsstr. 320B, 4052 Basel, Switzerland
^3^  Department of Oncology, University Hospital Basel, Petersgraben 4, 4031 Basel, Switzerland

**Background:** A sensorimotor training (SMT), mostly applied as balance training on different surfaces and in different positions, has the potential to contribute to the nervous system’s plasticity and to improve lower extremity impairments. In recent years, SMT has not only been conducted in rehabilitation, injury and fall prevention, but has also been successfully applied in adult oncology. In this context, studies showed improvements in common lower extremity impairments, such as impaired balance control, sensory and motor symptoms. While SMT or associated training modalities have been investigated in different pediatric patient collectives, they have rarely been conducted in pediatric oncology, though the outlined effects are promising for these patients. 

**Methods:** In order to identify therapeutic potentials of SMT for pediatric oncology, current data on SMT in different pediatric patient collectives are reviewed. Furthermore, to gather preliminary insights on a playful and child-specific SMT in pediatric oncology, first pilot studies during and after inpatient medical treatment in this field are conducted. 

**Results:** SMT resp.-related training modalities with various pediatric patients demonstrate potential effects on lower extremity parameters. The preliminary results in pediatric oncology indicate that a child-specific and playful SMT for children after cancer treatment is feasible. Motivating sensorimotor exercises are identified. Further study results on SMT performed during acute pediatric oncological therapy will be available and presented at the congress. 

**Conclusions:** The reviewed interventions in pediatric collectives, and preliminary study results in pediatric oncology in particular, suggest that SMT might be a promising and targeted training modality supplementing exercise therapy for children with cancer.

### 2.27. Dancing Can Improve Perceived Psychosocial Wellbeing and Coping during in-Patient Admissions on a Paediatric Cancer Ward

**Rachael Keating ^1^, Eilis O’Shea ^2^ and Susan Hurley ^3^**

^1^  Physiotherapy Department, Children’s Health Ireland at Crumlin, Dublin, Ireland
^2^  Occupational Therapy Department, Children’s Health Ireland at Crumlin, Dublin, Ireland
^3^  Music Therapy Department, Children’s Health Ireland at Crumlin, Dublin, Ireland

**Background:** The “Tuesday Boogie” is a quality improvement initiative which was developed to promote physical activity and enhance psychosocial wellbeing amongst in-patients, parents and staff on an in-patient paediatric cancer ward. It involves children, parents and staff engaging in a 15 min group dancing session and runs on a weekly basis. We conducted a service evaluation to assess the feasibility of the dance-based intervention and whether it influenced the psychosocial wellbeing of participants. 

**Methods:** Child, parent and staff questionnaires were designed by the research team to evaluate the intervention. Both quantitative (Likert, multiple choice, dichotomous) and qualitative open format questions were included. Data were collected on a voluntary, anonymous basis from June to July 2021.

**Results:** In total, 39 questionnaires were completed (*n* = 4 child, *n* = 9 parent, *n* = 26 staff). A total of 97% of respondents had taken part in the intervention with 100% reporting the intervention was worthwhile. Of the respondents, 95% felt taking part improved their mood and 92% reported it helped them to cope with being in hospital. Furthermore, 77% of respondents felt less worry or stress during the intervention. The following qualitative data were collected: C1 “It is nice to be happy and dance instead of worrying”, P3 “Changes the energy…real mood shifter and connector”, P9 “M gets a chance to have fun and to interact with other kids which is so rare these days”, S22 “Shows patients that not everything that happens in hospital is scary”. 

**Conclusions:** A dance-based intervention for children, parents and staff on an in-patient paediatric cancer ward is feasible and has numerous psychosocial benefits.

### 2.28. The Potential of Ambulatory Assessment for Exercise Oncology

**Anna Vogelsang ^1,2^, Corinna Meyer-Schwickerath ^3,4^, Joachim Wiskemann ^3,4^ and Markus Reichert ^1,5,6^**

^1^  Department of eHealth and Sports Analytics, Faculty of Sport Science, Ruhr-University Bochum, Bochum, Germany
^2^  Faculty of Humanities, MSH Medical School Hamburg, Hamburg, Germany
^3^  University of Heidelberg, Institute of Sports and Sports Sciences, Heidelberg, Germany
^4^  Department of Medical Oncology, National Center for Tumor Diseases (NCT), Heidelberg, Germany
^5^  Mental mHealth Lab, Department of Sport and Sport Science, Karlsruhe Institute of Technology, Karlsruhe, Germany
^6^  Department of Psychiatry and Psychotherapy, Central Institute of Mental Health, Medical Faculty Mannheim, University of Heidelberg, Heidelberg, Germany

**Background:** The growing population of childhood cancer survivors is at risk of developing late chronic conditions and premature mortality. Physical activity (PA) can mitigate physical and psychological late effects, but only if engaged in regularly over sustained time periods. PA sustainment, however, remains challenging due to daily and within-daily fluctuations in how survivors feel, with whom they interact and in which environments they live. Thus far, exercise psychology has mainly focused on macro-temporal processes unfolding over weeks and months. To promote sustainable PA engagement, micro-temporal processes (e.g., unfolding over minutes, hours, days) need to be studied. Therefore, this review introduces ambulatory assessment as a method to gain insights into dynamic behavioural processes as they unfold in survivors’ everyday lives. 

**Methods:** Ambulatory assessment (AA) describes a group of computer- or smartphone-assisted methods to study behavioural, biological, and psychological processes in survivors’ everyday lives near real time. In this review, we discuss the characteristics of AA and expand on its promise for researching micro-temporal behavioural processes and on its potential for exercise oncology, such as the prediction of critical phases of PA relapse. This is exemplified in ongoing projects, and we expand on future avenues where ambulatory interventions might benefit survivors’ tailored care.

**Conclusions:** Insights into dynamic behavioural processes as they unfold in everyday life may critically add to our understanding of sustainable PA and the development of individualised treatment where and when it is needed. Avenues for research, prevention and treatment will be discussed as well as the acceptability, compliance and ethical issues of AA. 

### 2.29. Factors Related to Rehabilitation Adherence in Childhood Cancer: A Review

**Lynn R. Tanner ^1,2^ and Erica N. Schorr ^1^**

^1^  School of Nursing, University of Minnesota, Minneapolis, MN, USA
^2^  Physical Medicine & Rehabilitation, Children’s Minnesota, MN, USA

**Background:** Children and adolescents with cancer receive rehabilitation interventions for the functional impacts of the disease and its corresponding treatment. Adherence to these interventions varies greatly. The purpose of this review was to identify the factors related to adherence in rehabilitation. 

**Methods:** A systematic review was conducted using Ovid Medline and CINAHL databases with search terms related to pediatric cancer, rehabilitation, and adherence. Study eligibility criteria included the following: English language, participants receiving a physical therapy, occupational therapy, speech-language pathology, cognitive or exercise intervention or service, mean age of ≤18 years old, and measurement of factors related to adherence. The PRISMA 2020 statement for reporting systematic reviews guided data synthesis. The study quality was assessed using the Joanna Briggs Institute Critical Appraisal Tools.

**Results:** The review included 13 studies providing rehabilitation interventions (exercise, yoga, walking, cognitive training, and vibration plate) to 283 children aged 3–19 years with adherence levels of 61–91% measured by session attendance. The majority (85%) of the studies comprised exercise interventions with 69% of the interventions being multifaceted in nature including aerobic, strengthening, and flexibility components. The factors related to adherence fell into three categories: (1) organizational; (2) condition-related; and (3) personal. Common barriers included fatigue, illness, time, family scheduling, and motivation. Facilitators included peer or caregiver support and supervision.

**Conclusions:** The existing literature on rehabilitation adherence focuses mostly on exercise interventions delivered by a multitude of health care professionals. More research is needed to improve understanding of the factors related to adherence to rehabilitation interventions and services in survivors of childhood cancer.

